# Composted organic manures modulated soil–microbe interactions that enhanced the growth of tobacco by improving rhizospheric microbial structure and soil nutrients

**DOI:** 10.1128/spectrum.02586-25

**Published:** 2026-04-16

**Authors:** Nuo Shi, Heng Yao, Tikun Zhang, Gaokun Zhao, Yuping Wu, Guanghai Zhang, Peng Zhou, Na Wang, Ge Wang, Guanghui Kong, Yongping Li, Yuxiang Bai

**Affiliations:** 1 Yunnan Academy of Tobacco Agricultural Sciences, Kunming, China; 2 Yunnan Agricultural University 12616, Kunming, Yunnan, China; 3 Yunnan Tobacco Company Pu'er City company, Pu'er, Yunnan, China; Yeungnam University, Gyeongsan, Gyeongbuk, Republic of Korea

**Keywords:** organic manures, stacking time, soil chemical properties, rhizosphere microorganisms, tobacco plant dry matter

## Abstract

Stacking, a pivotal step in the preparation of composted organic manures, plays a crucial role in organic matter decomposition, nutrient availability, and soil microbial diversity, with effects dependent on the stacking duration. However, the mechanisms through which organic manures with different stacking times influence crop growth and development via alterations in the soil environment remain poorly understood. This study employed a pot experiment with tobacco plants, applying conventional fertilizers alongside organic cottonseed meal manures subjected to various stacking times (0, 10, 20, and 30 days). The primary aim was to examine the impact of cottonseed meal organic manures on the microbial community structure, function, and chemical properties of tobacco-growing soil, as well as to assess dry matter accumulation in tobacco plants. Results revealed that, compared to conventional fertilizers alone, the addition of cottonseed meal organic manures significantly enhanced soil organic matter content, with the 20d-stacked manure showing the highest increase (68.94%). Additionally, the composition of the soil microbial community was modified, which has increased the relative abundance of carbon and nitrogen metabolic functions. Dry matter accumulation in tobacco plants increased by 13.66%, 25.83%, 31.65%, and 9.69% across the different stacking times. In conclusion, composted organic manures regulate the soil–microbe system, promoting tobacco growth by enhancing the microbial structure of rhizosphere soil and improving nutrient availability. Cottonseed meal organic manures subjected to 20d of composting exhibited the most comprehensive benefits. The enrichment of beneficial microbes like *Chryseobacterium* and *Humicola* (potential antagonists of soil pathogens) might contribute to the observed enhancement in soil organic matter and available nutrients. This improvement in soil quality led to a balanced nutrient environment, ultimately promoting dry matter accumulation in tobacco plants. These findings offer robust theoretical support for the application of composted organic manures.

**IMPORTANCE:**

This study is important because it elucidates the crucial mechanisms by which composted organic manures, specifically through the regulation of the soil–microbe system, promote crop growth. It provides key evidence that the stacking duration is a critical factor, identifying a 20-day (d) composting period for cottonseed meal manure as optimal for delivering comprehensive benefits: (i) Enhanced soil health: significantly increasing soil organic matter and improving nutrient availability. (ii) Improved microbial community: shifting the soil microbial structure to favor beneficial bacteria and suppress pathogens, thereby enhancing metabolic functions related to carbon and nitrogen cycling. (iii) Direct crop promotion: leading to a substantial increase in dry matter accumulation in tobacco plants

## INTRODUCTION

Oil meal, a by-product of oil extraction from grains and oilseeds such as soybeans, peanuts, and rapeseed, is a rich source of protein, dietary fiber, minerals, and other essential nutrients (1). The high organic matter content in oilseed-based manures enhances soil physical and chemical properties, boosting fertility and stimulating the growth of beneficial microorganisms. This process promotes root development, improves nutrient uptake, reduces susceptibility to root diseases, and ultimately supports the growth of aboveground plant structures (2). Cottonseed meal organic manure, a novel type of composted organic manure, is particularly rich in protein, amino acids, and free gossypol (3). This composition can enhance crop yield and quality, reduce fertilization costs, and increase economic returns, underscoring its significant potential.

Fertilization is crucial in agricultural practices (4), and adopting effective fertilization strategies is vital for sustainable crop production (5). Organic manures, derived from organic matter, encompass various types, including compost, green manure, oilcake manure, and biogas manure. Characterized by high organic matter content, a rich nutrient profile, and extended fertilization effects, organic fertilizers enhance soil fertility, improve the soil environment, regulate microbial community structures, and stimulate microbial metabolic activity over time (6). Suitable for long-term agricultural use (7), organic matter significantly improves crop quality compared to chemical fertilizers alone (8), making it indispensable for achieving high yield and quality (9). Moreover, organic manures have a more substantial impact on plant growth and yield formation than conventional fertilizers (10). The combined use of sunflower cake and flower cuttings, for example, increases sunflower height, stem circumference, and dry matter content while also enhancing soil fertility (11). Neem seed oil powder, another effective organic matter, reduces root-knot nematode populations and root nodule formation, while enhancing the length and weight of cucumber branches and fruits (12). Oilseed-based organic manures offer sustainable alternatives to mineral fertilizers, supplying essential nutrients for organic crop production. Repeated application of these organic materials not only improves soil organic nitrogen pools but also reduces nitrate leaching and nitrate content in plants, thereby improving food safety (13).

Organic compost, a vital type of organic manure, is produced through the partial high-temperature aerobic decomposition of animal and plant residues and excreta, transforming them into nutrient-dense material. Research indicates that the maturity of organic compost is essential for its efficacy, influencing soil bacterial diversity and richness, altering soil fertility, and affecting crop growth (14). Properly matured organic compost positively impacts soil improvement and yield enhancement, while fostering synergistic interactions among microorganisms (15). The maturity of organic compost is commonly assessed using several indicators (16), such as temperature, odor, color, volume, carbon-to-nitrogen (C/N) ratio, and seed germination rate. Initially, the temperature of the compost pile rises to a high phase before gradually declining. Upon maturation, the temperature stabilizes to a level consistent with or slightly above ambient temperature. Odorous compounds, including ammonia, dissipate as the compost matures. The compost’s color darkens, and its volume decreases to approximately one-half to two-thirds of its original size. The C/N ratio decreases from an initial value of 30 or higher to a range of 15–20. The seed germination index (GI) serves as an additional maturity indicator, with a GI greater than 50% signaling maturity and a GI exceeding 80% indicating high maturity. While numerous detailed and precise indicators for assessing organic compost maturity exist, they are often difficult for farmers to apply accurately in practical settings. Additionally, some farmers resort to directly applying unstacked organic manures in field production (17). Consequently, varying stacking times present a more intuitive and practical method for assessing compost maturity in actual production contexts. This study, therefore, aims to examine the effects of cottonseed meal organic manures with different stacking durations on soil nutrients in the rhizosphere of tobacco plants, dry matter accumulation in tobacco plants, and the microbial community structure in the rhizosphere soil. Furthermore, it aims to explore the pivotal role of the microbial community in the context of organic manure application, soil nutrient changes, and variations in tobacco plant dry matter content. These findings will provide a solid theoretical foundation for the scientific use of composted organic manures and contribute to the sustainable development of agriculture.

## MATERIALS AND METHODS

### Tobacco seedlings, soil preparation, and organic manures

The tobacco variety used in this experiment was Yunxue 39 (Nicotiana tabacum), sourced from the Yunnan Tobacco Research Institute in Yuxi, Yunnan Province, China. The test soil, also obtained from the same institute, had the following characteristics: pH 7.30, 1.84% organic matter, 0.15% total nitrogen, 0.15% total phosphorus, and 1.35% total potassium. According to the Chinese classification system for Quaternary Red Clay, the soil is classified as typical red soil. The previous crop was oilseed rape. Cottonseed meal organic manures were produced at the Tobacco Biochemistry Laboratory of Yunnan Agricultural University. After hot extraction of oil from cottonseed meal through hot pressing, the defatted cottonseed meal was thoroughly mixed with an organic material maturation agent (containing ≥0.5 billion effective viable bacteria per gram, Zhongnonglvkang, Beijing, China) at a ratio of 1 kg of fermentation starter to 300 kg of cottonseed meal. The mixture was then set aside for further processing. The prepared organic manure mixture was spread evenly onto a plastic sheet and formed into a pile measuring approximately 0.5–1 m in height, 1–2 m in width, and 2–3 m in length for composting. The pile was maintained at a temperature of 55°C to 60°C, with humidity levels kept between 65% and 70%. At 0, 10, 20, and 30 days (d) of stacking, five vertical samples were taken from the top to the bottom of the fermentation pile. These samples were thoroughly mixed to represent organic manures at different stacking times, and the resulting mixture was used in subsequent experiments. The basic properties of the cottonseed meal organic manures are provided in Tables 1 and 2.

**TABLE 1:** Nutrient composition of cottonseed meal organic manures at varying stacking times^*a*^

Treatment	pH	OM	TN	P_2_O_5_	K_2_O
		(%)	(%)	(%)	(%)
MZ0	6.11 ± 0.18a	68.14 ± 2.04ab	1.36 ± 0.04b	0.56 ± 0.02a	0.92 ± 0.03b
MZ1	5.48 ± 0.16b	64.75 ± 1.94b	1.48 ± 0.04a	0.48 ± 0.01b	1.03 ± 0.03a
MZ2	5.80 ± 0.17ab	70.91 ± 2.13a	1.33 ± 0.04b	0.40 ± 0.01c	0.89 ± 0.03b
MZ3	5.79 ± 0.17ab	69.74 ± 2.09a	1.25 ± 0.04c	0.31 ± 0.01d	0.75 ± 0.02c

^*a*^ Different letters show significant differences at *P* < 0.05.

**TABLE 2:** Microbial diversity profile of cottonseed meal organic manures at varying stacking times^*a*^

Treatment	Bacterial diversity					Fungal diversity				
	Sobs	Shannon	Simpson	Chao1	ACE	Sobs	Shannon	Simpson	Chao1	ACE
MZ0	501.67 ± 35.64a	2.68 ± 0.17b	0.69 ± 0.02b	547.30 ± 26.68b	568.93 ± 26.07b	696.33 ± 21.55a	3.09 ± 0.27ab	0.56 ± 0.05c	829.51 ± 41.04a	829.19 ± 52.00a
MZ1	589.00 ± 15.10a	5.05 ± 0.14a	0.93 ± 0.01a	649.36 ± 24.39a	673.06 ± 21.69a	485.00 ± 14.42c	3.32 ± 0.13a	0.83 ± 0.07a	668.95 ± 21.19b	671.37 ± 23.37b
MZ2	572.00 ± 70.17a	4.44 ± 0.51a	0.85 ± 0.06a	631.17 ± 74.46ab	649.61 ± 79.27ab	530.00 ± 142.06b	3.00 ± 0.22ab	0.68 ± 0.08b	697.75 ± 155.74b	701.40 ± 153.99b
MZ3	570.33 ± 43.66a	4.80 ± 0.51a	0.89 ± 0.05a	619.91 ± 42.20ab	637.00 ± 41.47ab	299.67 ± 155.85d	2.89 ± 1.46b	0.78 ± 0.10a	432.91 ± 113.12c	430.79 ± 125.59c

^*a*^ Stack organic manures without stacking; the Sobs index of bacteria increased by 17.41%, 14.02%, and 13.69%, respectively. Additionally, the Shannon index exhibited increases of 88.43%, 65.67%, and 79.10%. The Simpson index also rose by 34.78%, 23.19%, and 28.99%, while the Chao1 index increased by 18.65%, 15.32%, and 13.27%. Furthermore, the ACE index showed increases of 18.30%, 14.18%, and 11.96%. Among the fungal Sobs indices, MZ3 recorded the lowest value at 299.67, whereas the Shannon and Simpson indices for MZ1 were the highest at 3.32 and 0.83, respectively. Notably, the Chao1 and ACE indices for unstacked organic manures were both greater than those for stack organic manures. Different letters show significant differences at *P* < 0.05.

### Field site and experimental design

The experiment was conducted at the Yunnan Tobacco Research Institute in Yuxi (25°41′19″N, 103°26′13″E, altitude 1855 m), located in the Yunnan-Guizhou Plateau, which has a semi-humid, cold winter, mid-subtropical plateau monsoon climate. The region experiences an average annual temperature of 16.7°C, with annual precipitation ranging from 700 to 900 mm and average annual sunshine lasting between 2,300 and 2,700 h. An indoor pot experiment was performed with five treatment groups. The control group (CK) received conventional fertilization, while the treatment groups were exposed to organic manures made from unstacked cottonseed meal (MZ0) and cottonseed meal stacked for 10 days (MZ1), 20 days (MZ2), and 30 days (MZ3). In the control group, 2.50 g/plant of pure nitrogen was applied. In the organic manure treatments, 200 g/plant of organic manure from different stacking times was used, in addition to 2.50 g/plant of pure nitrogen. Each treatment included nine biological replicates. The organic manures were blended with soil (each pot containing 35 kg of soil) and left for 3 days to equilibrate before transplanting tobacco seedlings into the pots.

### Soil chemical property determination and dry matter content determination

At 60 days post-transplant, tobacco plants were uprooted with an adequate amount of soil and transferred to the laboratory in polyethylene bags. Non-rhizosphere soil was removed by gently shaking the roots, leaving only the strongly adhering rhizosphere soil. The tightly adhering rhizosphere soil was then separated from the roots by gently rubbing the surface of the roots (18). Soil samples were collected in duplicate for each repetition: one portion was stored at −80°C for microbial analysis, and the other was air-dried for analysis of pH, organic matter (OM), total nitrogen (TN), total phosphorus (TP), total potassium (TK), alkaline nitrogen (AN), and available potassium (AK). Soil pH was measured in a 1:2.5 soil (g)-to-water (mL) ratio (19). OM was determined using the potassium dichromate dilution heat method (20). TN was measured with a Kjeldahl nitrogen analyzer (multi N/C 2100 S, Analytik Jena, Germany) (21). TP was assessed using the acid-soluble molybdenum-antimony colorimetric method (22). TK was measured by NaOH alkali fusion and flame photometry (23). AN was determined by the alkaline hydrolysis diffusion method (24). Available phosphorus (AP) was measured using sodium bicarbonate extraction and molybdenum-antimony spectrophotometry (25) and available potassium (AK) by ammonium acetate extraction followed by flame photometry (26).

At 60 days post-transplant, the roots, stems, and leaves of each tobacco plant were thoroughly rinsed with distilled water, weighed for dry weight of roots, stems, and leaves, and dried at 105°C for 15 min before being further dried to a constant weight at 65°C (27). The root, stem, and leaf dry weights were recorded using an analytical balance.

### Amplicon sequencing analysis methods

#### DNA extraction

Total genomic DNA was extracted from the soil samples using the HiPure Soil DNA Kit (Magen, China) according to the manufacturer’s instructions. The integrity of the extracted genomic DNA was assessed by 1% agarose gel electrophoresis, and its concentration was measured using a Qubit 3.0 Fluorometer (Thermo Fisher Scientific, USA).

#### PCR amplification and sequencing library preparation

The extracted DNA was used as the template to amplify the target regions. The 16S rRNA V3–V4 region (primers 341F: 5′-CCTACGGGNGGCWGCAG-3′, 806R: 5′-GGACTACHVGGGTATTAAT-3′) and the ITS2 region (primers ITS3_KYO2: 5′-GATGAAGAACGYAGYRAA-3′, ITS4: 5′-TCCTCCGCTTATTGATATGC-3′) were amplified from total DNA using barcoded primers. The PCR reaction was performed in a 50 μL system containing the following: 10 μL of 5× Q5 Reaction Buffer, 10 μL of 5× Q5 High GC Enhancer, 1.5 μL of 2.5 mM dNTPs, 1.5 μL each of forward and reverse primers (10 μM), 0.2 μL of Q5 High-Fidelity DNA Polymerase, and approximately 50 ng of template DNA. All PCR reagents were purchased from New England Biolabs (USA). The thermal cycling conditions were as follows: initial denaturation at 95°C for 5 min; 30 cycles of denaturation at 95°C for 1 min, annealing at 60°C for 1 min, and extension at 72°C for 1 min; followed by a final extension at 72°C for 7 min. A negative control (PCR-grade water instead of template DNA) was included in each PCR run to monitor potential reagent contamination.

The PCR products were verified on a 2% agarose gel (with no band observed in the negative control), purified using AMPure XP Beads (Beckman Coulter, USA), and quantified with the Qubit 3.0 Fluorometer. Sequencing libraries were then constructed using the Illumina DNA Prep Kit (Illumina, USA). Finally, paired-end sequencing (2 × 250 bp) was performed on an Illumina NovaSeq 6000 platform.

### Bioinformatic analysis of high-throughput sequencing data

The raw paired-end reads were first quality-controlled using FASTP (version 0.18.0) (28) by the following: (i) removing reads containing ≥10% ambiguous bases (N); (ii) discarding reads containing adapters; and (iii) trimming low-quality bases (quality score ≤20) with the threshold that if over 50% of bases in a read were of low quality, the entire read was discarded. The remaining high-quality paired-end reads were merged into single sequences (tags) using FLASH (version 1.2.11) (29) with a minimum overlap of 10 bp and a maximum mismatch ratio of 0.2. These raw tags were further filtered following established criteria (30): tags were truncated at the first site where consecutive low-quality bases (quality score ≤3) reached a length of 3, and any tag whose length after truncation was less than 75% of the original length was discarded. The resulting high-quality sequences were designated as clean tags.

The clean tags were clustered into operational taxonomic units (OTUs) at a 97% similarity threshold using the UPARSE algorithm within USEARCH (version 11.0.667) (31). The most abundant sequence within each OTU was selected as its representative sequence. Chimeric sequences were detected and removed using the UCHIME algorithm (32). Taxonomic classification was performed using the RDP classifier (version 2.2) (33) against the SILVA 16S rRNA gene database (release 138.1) (34) for bacteria and the UNITE database (version 10.0) (35) for fungi, with a confidence threshold of 80%.

### Statistical analyses

All statistical analyses were performed using PASW Statistics 18 (SPSS Inc., Chicago, USA). The Shapiro-Wilk test assessed normality, and the F-test evaluated the homogeneity of variances before testing for significance. Significant differences among the samples were determined by one-way ANOVA, followed by Duncan’s multiple range test. The 16S rRNA gene and ITS amplicon sequencing data were processed using a comprehensive bioinformatics pipeline to profile the bacterial and fungal communities in the rhizosphere soil samples. Alpha diversity indices, including Shannon and Chao1, were calculated using the Omicshare platform (https://www.omicshare.com) to evaluate the richness and diversity of bacterial communities. Beta diversity analysis, employing principal coordinate analysis (PCoA) via the Omicshare platform, was conducted to assess the overall community composition and the similarities or dissimilarities between samples. The linear discriminant effect size (LEfSe) analysis was used to identify bacterial and fungal communities showing significant differences across samples, performing linear discriminant analysis (LDA) based on taxonomic composition and grouping conditions. Communities or differential microbes with absolute LDA scores > 2 and *P* < 0.05 were reported (36). To determine whether the addition of cottonseed meal organic fertilizer significantly impacted soil microbial assembly, co-occurrence networks were constructed using pairwise Spearman correlation analysis in R, focusing on the relative abundance of bacterial and fungal genera. Valid connections between nodes were defined as correlations with *P* ≤ 0.01 and |r| > 0.5, after Benjamini-Hochberg correction. The networks were visualized and analyzed in Gephi 0.9 to compare topological features across treatments. Bacterial functional predictions were annotated using the KEGG pathway in PICRUSt2 and FAPROTAX, while fungal functions were annotated using FUNGuild. Partial least squares path modeling (PLS-PM) with the “plspm” package in R (version 3.4.3) was applied to evaluate the relationships between maturation time, the basic properties of cottonseed meal organic fertilizer (nutrient content and microbial diversity), soil physicochemical characteristics, bacterial and fungal alpha diversity, bacterial and fungal differential microbes, and dry matter content. Path coefficients and R^2^ values were estimated and validated through bootstrap methods (10,000 iterations), excluding latent variables with loading values below 0.7 to maintain model validity. The overall predictive performance of the model was assessed using Goodness of Fit (GOF) indicators (37).

## RESULTS

### Effects of cottonseed meal organic manure application at different stacking times on soil nutrient content and tobacco dry matter

**Fig 1:**
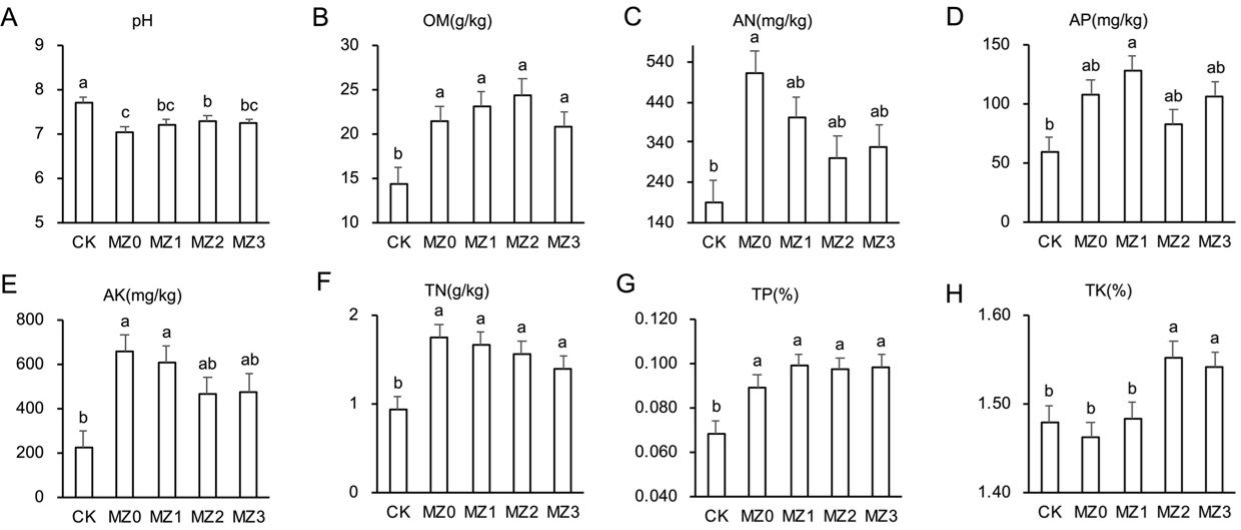
Effects of cottonseed meal organic manures with varying stacking times on soil nutrient content. The parameters measured include pH (**A**), organic matter (OM) (**B**), alkaline nitrogen (AN) (**C**), available phosphorus (AP) (**D**), available potassium (AK) (**E**), total nitrogen (TN) (**F**), total phosphorus (TP) (**G**), and total potassium (TK) (**H**).

Soil chemical properties 60 days post-tobacco transplantation are illustrated in Fig. 1. Organic manure treatments significantly reduced pH compared to CK, with decreases of 8.53%, 6.48%, 5.21%, and 6.07%, respectively (Fig. 1A). The OM, TN, and TP contents were substantially higher in the organic manure treatments, increasing by 48.52%, 60.10%, 68.94%, and 43.40% (Fig. 1B); 85.61%, 77.71%, 66.97%, and 49.00% (Fig. 1F); and 30.46%, 44.16%, 41.77%, and 43.28% (Fig. 1G), respectively. Furthermore, AN in MZ0 increased by 164.61% (Fig. 1C), AP in MZ1 by 116.29% (Fig. 1D), and AK in MZ0 and MZ1 by 196.12% and 172.24%, respectively (Fig. 1E). TK in MZ2 and MZ3 increased by 4.97% and 4.20%, respectively, compared to CK (Fig. 1H).

**Fig 2:**
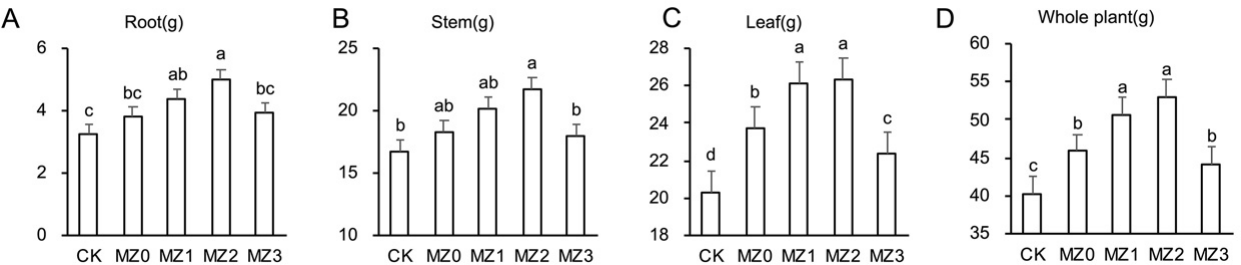
Effects of cottonseed meal organic manures with varying stacking times on dry matter content in cigar tobacco, including the root (**A**), stem (**B**), leaf (**C**), and whole plant (**D**).

Tobacco plant dry matter accumulation 60 days after transplantation is presented in Fig. 2. Root dry weight in MZ1 and MZ2 increased significantly by 34.69% and 53.06%, respectively, compared to CK (Fig. 2A). Stem dry weight in MZ2 increased by 30.08% compared to CK (Fig. 2B). Leaf dry weight in the organic manure treatments showed significant increases of 16.61%, 28.44%, 29.51%, and 9.89% (Fig. 2C), and total dry weight increased by 13.66%, 25.83%, 31.65%, and 9.69% (Fig. 2D).

### Effects of cottonseed meal organic manure application at different stacking times on soil microbial community structure

#### Microbial diversity analysis

##### Microbial α-diversity

**Fig 3:**
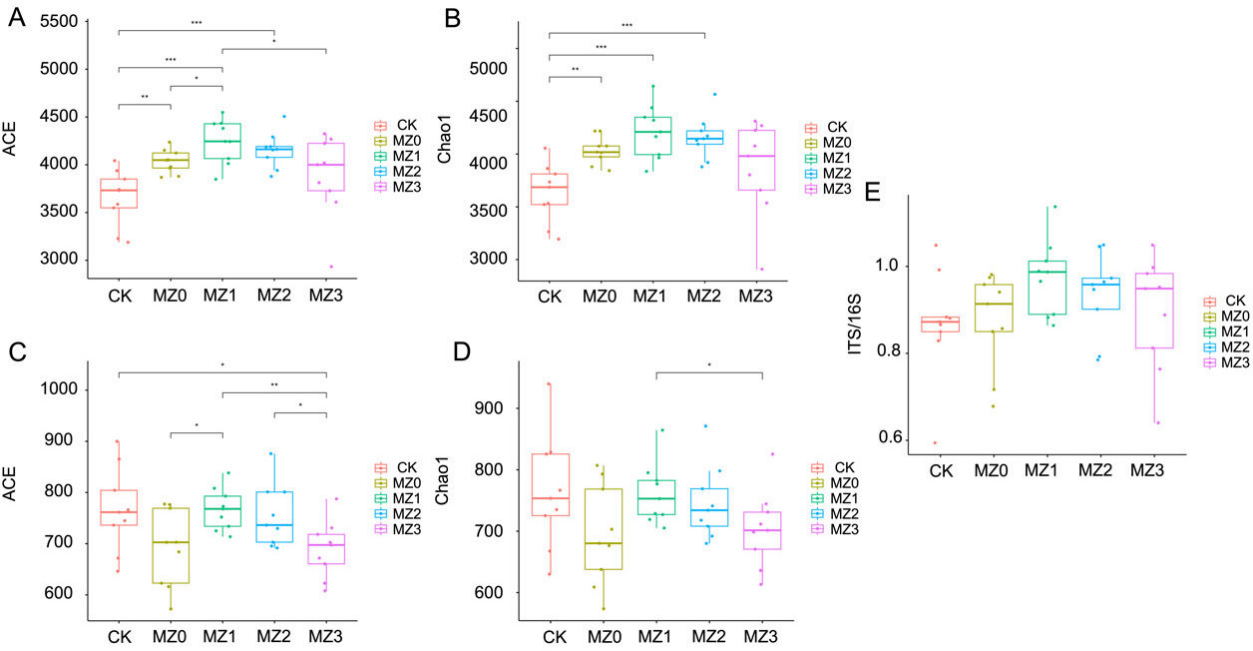
Effects of cottonseed meal organic manures with varying stacking times on soil bacterial and fungal alpha diversity. The figures depict the ACE index of bacteria (**A**), Chao1 index of bacteria (**B**), ACE index of fungi (**C**), Chao1 index of fungi (**D**), and the ITS/16S ratio (**E**).

Changes in microbial community indices in response to organic manure application are shown in Fig. 3. The ACE and Chao1 indices for soil bacteria initially increased, reaching their peak in the MZ1 treatment before declining, suggesting that bacterial diversity peaked in MZ1, while community richness was highest in MZ3. For soil fungi, both indices (Fig. 3C and D) followed a similar trend, with peak diversity and richness observed in MZ2 and MZ3, respectively. In the CK and MZ0, the fungal-to-bacterial ratio (ITS/16S) gradually decreased, while it significantly increased in MZ1, MZ2, and MZ3 (Fig. 3E). Compared with the control group, the application of organic fertilizers increased ITS/16S by 0.69%, 12.20%, 7.69%, and 2.81% in the MZ1, MZ2, and MZ3 treatments, respectively.

##### Microbial β-diversity

**Fig 4:**
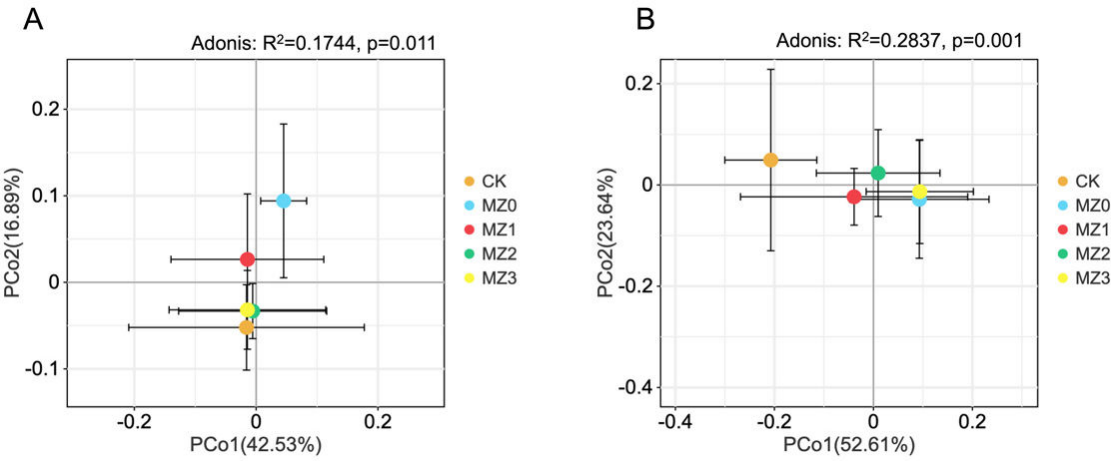
Beta diversity of soil bacterial and fungal communities influenced by cottonseed meal organic manures with varying stacking times. Principal coordinate analysis (PCoA) maps represent bacterial communities (**A**) and fungal communities (**B**).

Principal coordinate analysis (PCoA) results revealed that the soil bacterial community data from different treatments were primarily distributed along the first two principal components. The first axis accounted for 42.53% of the variation, while the second axis explained 16.89%, with a cumulative variance contribution rate of 59.42% (Fig. 4A). Notably, significant differences were observed between the bacterial communities in the MZ0 and MZ1 treatments, while those in the MZ2 and MZ3 treatments showed less distinction. The separation between the organic manure treatments was evident (R^2^ = 0.1744, *P* = 0.011), indicating that the stacking time of the organic manures significantly influenced the diversity of rhizosphere bacteria in tobacco. Similarly, the soil fungal community data were primarily concentrated along the first two principal components, with the first axis contributing 52.61% and the second 23.64%, resulting in a cumulative variance contribution rate of 76.25% (Fig. 4B). In contrast to the bacterial communities, the fungal communities showed distinct separation following the application of organic manures at different stacking times. Fungal communities treated with MZ0 and MZ3 clustered together (R^2^ = 0.2837, *P* = 0.001). These results demonstrated that the stacking time of organic manures also had a notable impact on the diversity of rhizosphere fungi in tobacco.

### Microbial composition analysis

**Fig 5:**
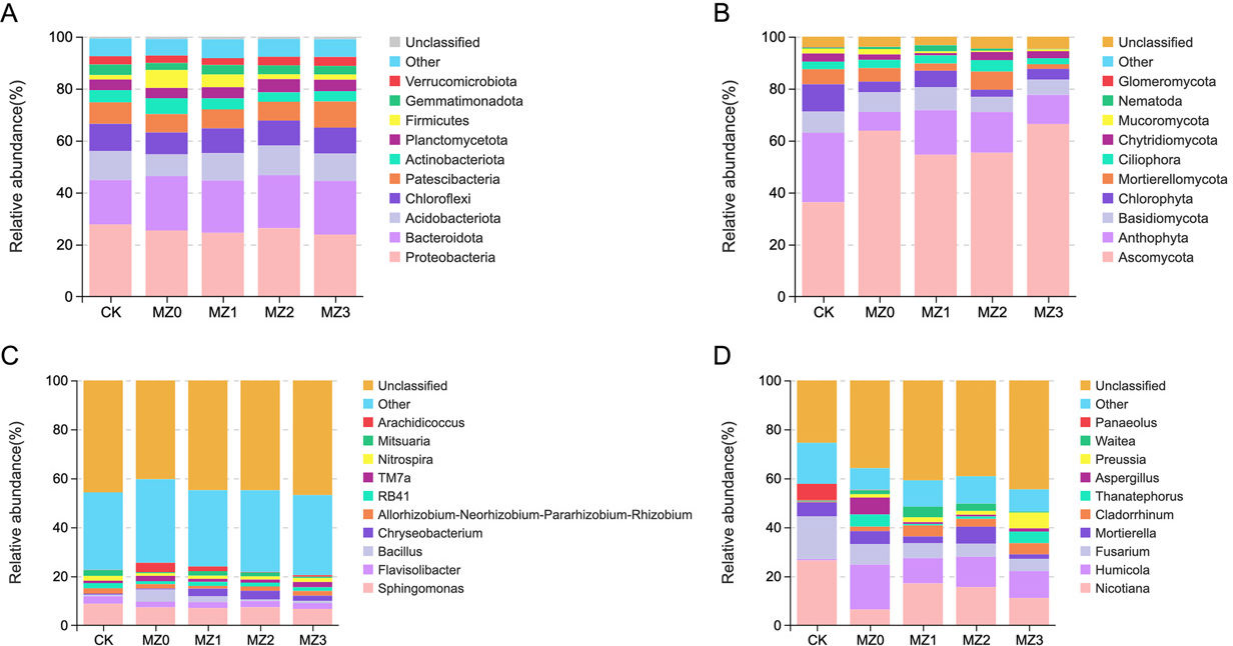
Comparison of bacterial and fungal communities under treatments CK, MZ0, MZ1, MZ2, and MZ3. Panels depict the relative abundance of bacterial phyla (**A**), fungal phyla (**B**), bacterial genera (**C**), and fungal genera (**D**).

Significant differences in the relative abundance of dominant bacterial phyla across treatments are shown in Fig. 5A. The five most abundant bacterial phyla are Proteobacteria, Bacteroidota, Acidobacteriota, Chlorophyta, Chloroflexi, and Patescibacteria. While Proteobacteria did not exhibit notable variation among treatments, its highest abundance was observed in the MZ2 and MZ3 treatments. Compared to conventional fertilization (CK), organic manure applications increased the relative abundance of Bacteroidota and Acidobacteriota by 3.05% to 3.75% and 2.09% to 2.99%, respectively. Additionally, compared to the MZ0 treatment, the relative abundance of Chloroflexi and Patescibacteria in the other organic manures treatments increased by 1.16% to 1.59% and 0.24% to 2.97%, respectively.

Differences in the relative abundance of dominant fungal phyla across treatments are shown in Fig. 5B. The five most abundant fungal phyla are Ascomycota, Anthophyta, Basidiomycota, Chlorophyta, and Mortierellomycota. Following the application of cottonseed meal organic manures, the relative abundance of Ascomycota increased by 18.27% to 30.14% compared to CK, while the abundances of Anthophyta and Chlorophyta decreased by 9.62% to 19.57% and 4.16% to 7.75%, respectively.

Figure 5C shows notable differences in the relative abundance of dominant bacterial genera across treatments. In comparison to CK, the relative abundances of *Sphingomonas* and *Flavisolibacter* remained relatively stable following organic manure application. However, the relative abundances of *TM7a*, *Chryseobacterium*, and *Bacillus* increased by 0.04% to 3.09%, 0.07% to 4.25%, and 0.27% to 1.21%, respectively.

It is shown in Fig. 5D that there were significant differences in the relative abundance of dominant fungal genera across treatments. Compared to CK, organic manure application resulted in increased relative abundances of *Humicola* and *Cladorrhinum*, with increases of 9.79% to 17.79% and 1.55% to 4.26%, respectively. Meanwhile, the relative abundance of *Fusarium* decreased by 8.96% to 12.33%.

### LEfSe analysis

Linear discriminant effect size (LEfSe) analysis, with a predefined linear discriminant analysis (LDA) score threshold, was employed to assess differences in microbial abundance and their effects between groups. The LEfSe results (LDA > 2, *P* < 0.05) revealed significant differences across treatments, identifying a total of 169 differential bacterial microorganisms (or microbial taxa) and 125 differential fungal microorganisms (or microbial taxa) from the phylum to the family level (Tables S1 and S2). At the family level, the CK treatment exhibited 22 differential bacterial taxa. In contrast, the MZ0, MZ1, MZ2, and MZ3 treatments displayed 59, 17, 42, and 29 differential bacterial taxa, respectively. For fungi, the CK treatment presented 71 differential taxa at the family level, while the MZ0, MZ1, MZ2, and MZ3 treatments had 9, 18, 13, and 14 differential taxa, respectively. These results suggest that the application of decomposed organic manure significantly enriched soil bacterial genera compared to conventional fertilizer, whereas the fungal community did not show a comparable response.

### Co-occurrence network analysis

**Fig 6:**
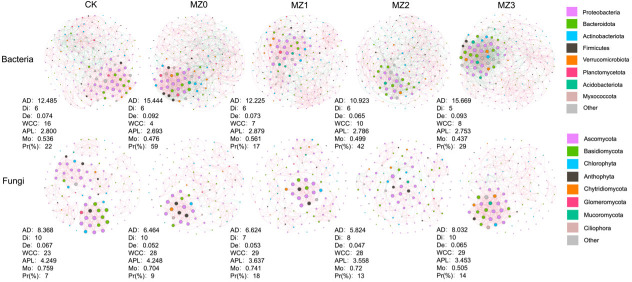
Symbiotic network topology of bacterial and fungal communities influenced by cottonseed meal organic manures with varying stacking times. Node colors represent microbial phyla: purple for Proteobacteria, green for Bacteroidota, blue for Actinobacteriota, black for Firmicutes, orange for Verrucomicrobiota, pink for Planctomycetota, cyan for Acidobacteriota, light brown for Myxococcota, and gray for other phyla. Nodes represent microbial genera. Red lines indicate positive correlations between genera, while green lines represent negative correlations. Topological properties in the co-occurrence network include Average Degree (AD), Diameter (Di), Density (De), Weakly Connected Components (WCC), Average Path Length (APL), Modularity (Mo), and Proportion (%) (Pr).

A co-occurrence network of the differential microbial taxa, based on the LEfSe results, was constructed to illustrate microbial interactions at both the phylum and genus levels (Fig. 6). The complexity of the bacterial and fungal community networks shifted considerably with organic manure application at various decomposition stages. The topological parameters revealed that the bacterial network exhibited a higher Average Degree and Density, whereas the fungal network had larger values for Diameter, Weakly Connected Components, Average Path Length, and Modularity. Organic manure application increased the proportion of soil bacteria while reducing the proportion of fungi, indicating that it enhances the stability of the soil microbial community structure. In the bacterial networks of each treatment, the biased enrichment circle highlights core microorganisms with an Eigenvector Centrality greater than 0.5. These core microorganisms mainly belong to the phyla Proteobacteria and Bacteroidota. The number of core genera was 29 in CK, 36 in MZ0, 27 in MZ1, 24 in MZ2, and 40 in MZ3. Key bacterial genera identified in the organic manure treatments include *Aridibacter*, *DSSF69*, *Parapedobacter*, *Larkinella*, and *Chryseobacterium*. Similarly, in the fungal network, the core microorganisms primarily belong to the phyla Ascomycota and Basidiomycota. The core fungal genera were 36 in CK, 17 in MZ0, 15 in MZ1, 16 in MZ2, and 26 in MZ3. Among the fungal core genera identified in the cottonseed meal treatments, key genera included *Coelastrella*, *Candida*, *Ustilago*, *Stephanonectria*, and *Humicola*.

### Functional prediction

**Fig 7:**
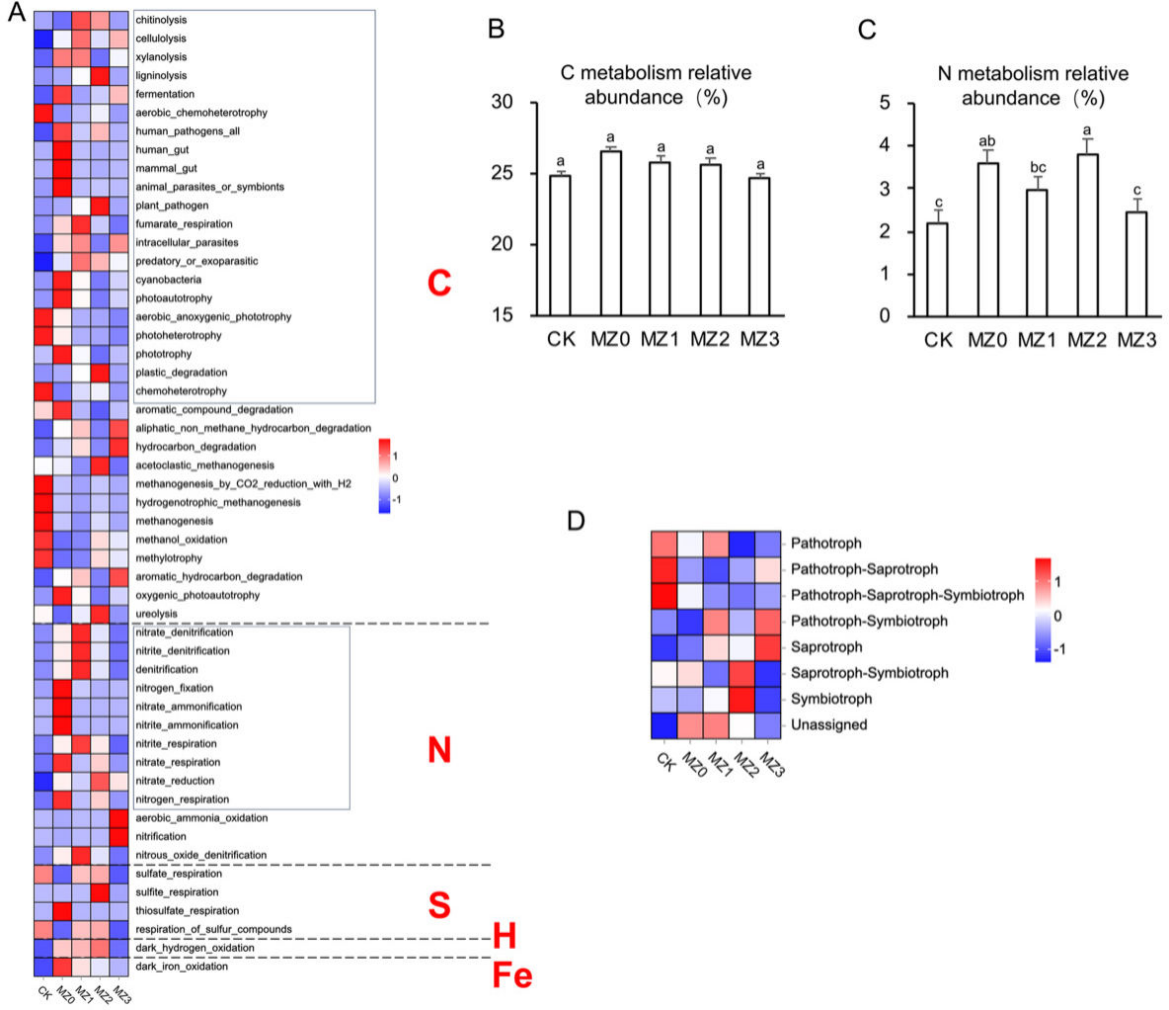
Heat map of soil microorganism functional diversity under different treatments. The color depth indicates the variation between the expression of metabolic functions and the mean. The average gene expression level of the same sample serves as the baseline, with positive values (higher than average expression) shown in red and negative values (below average expression) marked in purple. (**A**) Heat map of bacterial predictive function. (**B and C**) Relative abundance of C and N metabolic functions, specifically highlighting the abundance of C and N metabolism in the rectangular frame from Figure (7A), focusing solely on C and N elements. (**D**) Heat map of fungal predictive function.

Functional prediction analyses using PICRUSt2 and FAPROTAX indicated that soil bacterial communities across treatments were enriched in the metabolism of C, N, S, H, Fe, and other elements, with carbon and nitrogen metabolic functions being particularly prominent. Organic manure application significantly altered the functional characteristics of the soil bacterial community compared to CK (Fig. 7A). Specifically, C metabolic functions in MZ1, MZ2, and MZ3 increased by 7.17%, 4.11%, and 3.41%, respectively (Fig. 7B), while N metabolic functions in MZ0, MZ1, MZ2, and MZ3 increased by 65.77%, 35.60%, 75.63%, and 11.81%, respectively (Fig. 7C). Fungal functional prediction using FUNGuild (Fig. 7D) revealed that the dominant functional groups were Pathotroph, Pathotroph-Saprotroph, Pathotroph-Saprotroph-Symbiotroph, Pathotroph-Symbiotroph, Saprotroph, Saprotroph-Symbiotroph, and Symbiotroph. Organic manure application enhanced the functions of Pathotroph-Symbiotroph and Saprotroph over time. Notably, MZ1 and MZ2 significantly promoted both Saprotroph-Symbiotroph and Symbiotroph functions, while MZ3 was more effective in enhancing Pathotroph-Symbiotroph and Saprotroph functions.

### Structural equation model analysis

**Fig 8:**
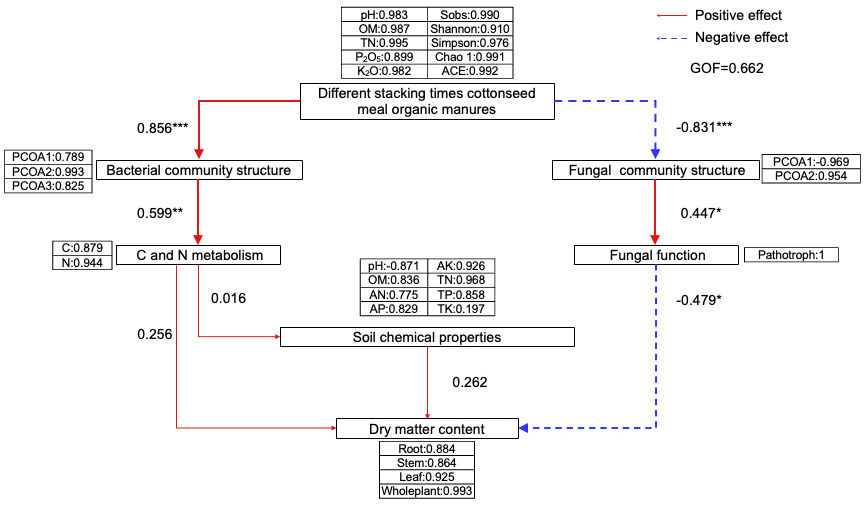
Structural equation model (SEM) depicting the relationships among different stacking times, cottonseed meal organic manures, the nutrient content of cottonseed meal organic manures, microbial diversity of cottonseed meal organic manures, microbial community structure, microbial function, soil chemical properties, and dry matter content. The arrow thickness corresponds to the strength of the path coefficient, with red arrows representing positive coefficients and purple arrows indicating negative coefficients. Path coefficients are denoted by “R,” and significance levels are marked by the following: * (*P* < 0.05), ** (*P* < 0.01), and *** (*P* ≤ 0.001). Different decomposed organic fertilizers include basic parameters such as pH, OM, TN, K_2_O, P_2_O_5_, and microbial diversity. Bacterial and fungal community structures are represented by PCoA. Soil chemical properties include pH, OM, AN, AP, AK, TN, TP, and TK.

A structural equation model (SEM) (Fig. 8) was constructed to examine the relationships among different stacking times of cottonseed meal organic manures, soil chemical properties, bacterial and fungal community structures, C and N metabolic functions, fungal functions, and dry matter accumulation. The model showed a good fit, with a GOF score of 0.662. The application of cottonseed meal organic manures at different stacking times positively influenced the bacterial community structure (r = 0.856) while negatively affecting the fungal community structure (r = −0.831). Both bacterial and fungal communities contributed to microbial functions in the soil (r = 0.599 for bacteria, r = 0.447 for fungi). Notably, bacterial C and N metabolic functions positively impacted soil chemical properties (r = 0.016) and positively impacted dry matter accumulation (r = 0.256). Soil chemical properties positively impacted dry matter accumulation (r = 0.262). Conversely, fungal functions had a significant negative effect on dry matter accumulation (r = −0.479). Overall, the application of cottonseed meal organic manures at different stacking times substantially influenced soil microbial communities and chemical properties, thereby affecting dry matter accumulation in plants.

## DISCUSSION

Organic manure treatments with varying stacking times led to an initial increase in OM content, which then declined, peaking at MZ2. TN content consistently decreased with extended stacking times, while TP gradually increased and stabilized. TK significantly increased in MZ2 and MZ3 treatments. All organic manure treatments improved the availability of soil nutrients, with MZ2 exhibiting the highest levels of available nutrients and OM content among the treatments. These results align with previous findings indicating that longer stacking times of organic manure tend to reduce soil organic matter content (15). Organic manure application enhances soil nutrient availability (38) and notably impacts TN, TP, and TK levels (39). However, excessive application of organic manure with extended stacking times should be avoided to prevent organic matter loss, optimize nutrient release, and maintain soil organic matter levels (40).

Changes in soil nutrient levels are closely linked to modifications in the soil microbial community. Organic manure, as a nutrient source, alters microbial community structure while providing carbon, nitrogen, and energy to microorganisms in the tobacco rhizosphere (41). In this study, the application of cottonseed meal organic manures with varying stacking times induced changes in the microbial community structure in the tobacco rhizosphere. Specifically, as the stacking time increased, soil bacterial diversity and richness initially rose and then declined. Similarly, the α-diversity of soil fungi showed an initial increase followed by a decrease. In this study, the application of cottonseed meal organic manures with different stacking times led to changes in the microbial community structure in the tobacco rhizosphere. Specifically, as the stacking time increased, the diversity and richness of soil bacteria initially rose but then decreased. Similarly, the α diversity of soil fungi initially increased but then decreased. The ITS/16S ratio first increased and then decreased, indicating that the proportion of fungi in the soil was higher than that of the bacterial community. The well-stacked organic manure provided a substrate type more suitable for fungi and created a microenvironment more conducive to fungal growth, ultimately allowing the fungal community that was more advantageous in resource competition to take the dominant position. This is because the application of organic fertilizers and other substances alters the flow of carbon sources, causing a change in the ratio of microorganisms (fungal-to-bacterial ratio) and also influencing the microbial carbon-nitrogen cycle. This functional transformation, in turn, enhances or weakens the community’s resistance and resilience to subsequent disturbances. β-diversity analysis further confirmed that cottonseed meal organic manures significantly reshaped the microbial community structure in the tobacco rhizosphere. Previous studies have reported significant shifts in microbial populations when organic and inorganic treatments are combined, notably a decrease in fungal abundance and an increase in bacterial abundance (42). The combined application of cow manure and vegetable oil meal promoted beneficial microorganisms in the rhizosphere, enhancing soil microbial metabolic activity (2). Similarly, humic acid fertilizer application altered the soil bacterial community by increasing the abundance of beneficial microorganisms like Actinomycetes and Firmicutes, while reducing harmful microorganisms such as Acidobacteria and Monastida (43). The use of organic fertilizers led to a bacterial-dominated microbial community, where *Bacillus* subtilis, introduced through manure, played a key role in promoting beneficial microbial colonization. Additionally, bio-organic manures boosted the metabolism and uptake of mineral elements while increasing the relative abundance of saprophytic bacteria (7). These effects can be attributed to the differential responses of soil microorganisms to organic manures. As the composting time increases, bacterial richness and structural diversity in the tobacco rhizosphere soil increase, while fungal richness and diversity decrease, favoring the enrichment of beneficial bacteria (44).

The application of cottonseed meal organic manures with varying stacking times significantly influenced soil microbial community structure, altering the relative abundance of different microbial groups. The results of this study revealed that organic manure treatments led to a gradual increase in the relative abundance of Acidobacteriota and Chloroflexi in the soil bacterial community. In contrast, the MZ2 and MZ3 treatments were associated with a higher relative abundance of Bacteroidetes. Previous studies have shown that Acidobacteriota plays a key role in nitrogen cycling, including ammonification, nitrification, and biological nitrogen fixation, as well as in sulfur cycling through processes such as sulfate reduction, sulfur oxidation, and sulfide oxidation, thus affecting both N and S cycles (45). Chloroflexi, a dominant group in soil and compost, actively participates in the cycling of carbon, nitrogen, and sulfur through processes (46) such as CO_2_ fixation, CO oxidation, CH_4_ oxidation, and the degradation of cellulose and other complex organic compounds. Bacteroidetes, recognized for breaking down high molecular weight polymers, play a vital role in carbon and nitrogen cycling and contribute to phosphorus cycling by converting insoluble phosphorus into soluble forms (47). Moreover, Bacteroidetes degrade plant and fungal residues in the soil, making them more accessible for plant growth and development (48). In the soil, both Bacteroidetes and Chloroflexi enhance carbon and nitrogen cycling by mineralizing and humifying plant and fungal residues, thereby increasing soil organic matter and improving soil fertility. In this study, the application of cottonseed meal organic manure with varying stacking times increased the relative abundance of Ascomycota in the soil fungal community. The MZ1 and MZ2 treatments stabilized the overall abundance of the fungal community, while the relative abundance of Basidiomycota increased. On the contrary, the MZ3 treatment led to a decrease in the relative abundance of Basidiomycota. It is notable that Ascomycota and Basidiomycota, as the two largest groups in the fungal kingdom, have highly diverse functions, covering various lifestyles from plant pathogens and animal pathogens to saprophytic decomposers and symbiotic bacteria (49). Previous studies have shown that the Chaetomiaceae family under the Ascomycota phylum can accumulate in high-temperature composting environments and decompose cellulose and chitin, while the Hypocreaceae family not only functions as decomposers but also acts as biocontrol agents to inhibit soil-borne pathogens, indirectly promoting plant health. The decrease in the abundance of Ceratobasidiaceae may be related to the antagonistic substances produced during the composting process and competitive microorganisms that inhibit them, and the abundance of Pleurotaceae, which has lignin-degrading ability, also decreased (50). This indicates that Ascomycota and Basidiomycota are closely related to manure stacking.

*Aridibacter* is known to influence cellular motility and signal transduction (51), while *DSSF69* and *Parapedobacter* are commonly found in compost (52). *Coelastrella* is recognized for its soil remediation properties (53), and both *Larkinella* and *Candida* are closely associated with organic fertilizer fermentation (54). *Ustilago*, a pathogenic fungus, is responsible for causing plant diseases, whereas *Stephanonectria* acts as an endophyte in plant roots (55). Notably, *Chryseobacterium* and *Humicola* emerged as the predominant bacterial and fungal genera, respectively, under the MZ2 treatment, with Eigenvector Centrality values exceeding 0.5. These strains have demonstrated the ability to promote plant growth, enhance stress tolerance, suppress soil pathogens, limit the proliferation of pathogenic fungi, and bolster plant resistance. The application of cottonseed meal organic manures with varying stacking times significantly increased the relative abundance of C and N cycling-related metabolic functions in the soil bacterial community, as predicted by functional analysis. These observations are consistent with those reported by Huang et al. (56), indicating that cottonseed meal organic manure treatments elevate carbon and nitrogen metabolism as critical functional processes within soil microbial communities. For fungal communities, the relative abundance of saprotrophic organisms was consistently higher across all treatments. The treatment of cottonseed meal organic manure has led to the emergence of various functional characteristics in the fungal community in the soil. The organic manures of MZ1 and MZ2 provided nutrients for saprophytic fungi, enabling plants to interact with symbiotic fungi, thereby simultaneously enhancing the functions of Saprotroph and Symbiotroph. The organic fertilizer of MZ3 provided abundant nutrients, stimulating the rapid reproduction of pathogenic bacteria. However, due to the excessive nutrients, it weakened the interaction between plants and symbiotic fungi, potentially enhancing the Pathotroph function (57, 58). It indicates that the functions of Saprotroph and Symbiotroph show a trend of increasing first and then decreasing with the increase in stacking time, and an appropriate composting time is beneficial for the increase in Pathotroph. The accumulation of dry matter in tobacco plants reflects the impact of cottonseed meal organic manure with varying stacking times on soil microbial community structure and nutrient dynamics. This study demonstrated that MZ1 and MZ2 treatments promoted dry matter accumulation in tobacco roots, stems, and leaves, while MZ0 and MZ3 treatments inhibited overall dry matter accumulation. Notably, the highest dry matter accumulation occurred under the MZ2 treatment, which was closely linked to improvements in the soil micro-ecological environment and nutrient availability. Under MZ2 treatment, microbial diversity and richness significantly increased, with higher abundances of dominant phyla such as Acidobacteriota, Chloroflexi, Bacteroidetes, Ascomycota, and Basidiomycota, all contributing to enhanced soil fertility. Furthermore, the presence of plant growth-promoting traits (PGPTs) in Acidobacteriota, along with the transformation of key plant nutrients, likely facilitated improved plant growth (56). *Chryseobacterium* and *Humicola*, key genera under MZ2 treatment, played pivotal roles in promoting plant growth and enhancing plant stress tolerance (59). The abundance of N metabolism functions was significantly higher under MZ2 treatment, likely due to the conversion of soil organic nitrogen from amino acids, which certain soil bacteria use as precursors in the synthesis of plant growth regulators, thus stimulating plant growth (60).

The model path indicates that the application of organic manure may directly enrich the nutrient reservoir in the soil and simultaneously cause changes in the microbial community in the soil. These two factors jointly affect the accumulation of dry matter in tobacco. It is notable that this influence shows different patterns in the bacterial and fungal communities: it seems to directly promote the improvement in soil chemical properties and the benign succession of the bacterial community, but it shows a negative correlation with the changes in the structure of the fungal community. We speculate that this may lead to a functional transformation: enhanced bacterial metabolism (such as carbon and nitrogen cycles) directly affects the growth of tobacco plants and may also have a positive effect on soil fertility improvement, thereby indirectly influencing the growth of tobacco plants, while the changes in the fungal community may not be beneficial for tobacco growth. However, it must be emphasized that the soil-plant-microbe system is a complex feedback loop network. The path revealed by the SEM model is a statistically supported correlation, rather than a definite unidirectional causal relationship. For example, plant root exudates also reverse select the microbial community, and their health status also affects the rhizosphere environment.

Although this study revealed the potential mechanism by which the composting time of cottonseed meal affects crop growth through microbial mediation under controlled conditions, there are still several limitations that need to be explored in future research. (i) This study was only verified in one crop (tobacco) and one soil type. The generalizability of its conclusion in different crop-soil systems needs to be further evaluated. (ii) Although greenhouse pot experiments can effectively control variables, they cannot fully simulate the complex climatic factors, pest and disease pressures, and interactions with other organisms in the field environment. Future research needs to be conducted under field conditions to more comprehensively reveal the mechanism.

### Conclusion

In this experiment, compared to conventional fertilizers, cottonseed meal organic manures with varying stacking times significantly altered the composition of soil microbial communities, enhanced soil nutrient levels, and boosted dry matter accumulation in tobacco plants. Among the treatments, MZ2 exhibited the most pronounced effect, markedly increasing soil microbial richness and diversity. Functional predictions revealed an elevated abundance of N metabolic functions, with the dominant genera *Chryseobacterium* and *Humicola* playing pivotal roles in maintaining soil microecological health and enhancing soil fertility. Moreover, MZ2 treatment resulted in the highest levels of soil organic matter and dry matter accumulation in tobacco plants. Therefore, the application of cottonseed meal organic manure under the MZ2 stacking time effectively promoted soil microbial diversity, improved soil chemical properties, and increased dry matter accumulation in tobacco plants. These findings provide a scientific basis for optimizing stacking times of organic manures in agricultural practices.

## Data Availability

The raw sequence data reported in this paper have been deposited in the Genome Sequence Archive (Genomics, Proteomics, & Bioinformatics, 2021) in National Genomics Data Center (Nucleic Acids Research, 2022), China National Center for Bioinformation/Beijing Institute of Genomics, Chinese Academy of Sciences (GSA: CRA022208) that are publicly accessible at https://ngdc.cncb.ac.cn/gsa.

## SUPPLEMENTAL MATERIAL

Supplemental tables spectrum.02586-25-s0001.docx Tables S1 and S2.

ASM does not own the copyrights to Supplemental Material that may be linked to, or accessed through, an article. The authors have granted ASM a non-exclusive, world-wide license to publish the Supplemental Material files. Please contact the corresponding author directly for reuse.

## References

[1] Ferchichi N, Toukabri W, Hammami I, Guiga C, Ajengui A, Mselhi W, Ben-Hammouda T, Bahri H, Trabelsi D. 2023. Valorization of oil cakes as a soil amendment for wheat cultivation through laccase-producing bacteria Bacillus pumilus. J Soil Sci Plant Nutr 23:6101–6113. doi:10.1007/s42729-023-01467-1

[2] Chen YX, Lv XL, Qin YM, Zhang DP, Zhang CQ, Song ZF, Liu DY, Jiang LQ, Huang B, Wang J. 2023. Effects of different botanical oil meal mixed with cow manure organic fertilizers on soil microbial community and function and tobacco yield and quality. Front Microbiol 14:1191059. doi:10.3389/fmicb.2023.1191059 37303792 PMC10248155

[3] Świątkiewicz S, Arczewska-Włosek A, Józefiak D. 2016. The use of cottonseed meal as a protein source for poultry: an updated review. Worlds Poult Sci J 72:473–484. doi:10.1017/S0043933916000258

[4] Tröster MF, Pahl H, Sauer J. 2019. Effects of application costs on fertilizer application strategy. Comput Electron Agric 167:105033. doi:10.1016/j.compag.2019.105033

[5] Xiang XJ, Liu J, Zhang J, Li DM, Xu CX, Kuzyakov Y. 2020. Divergence in fungal abundance and community structure between soils under long-term mineral and organic fertilization. Soil Tillage Res 196:104491. doi:10.1016/j.still.2019.104491

[6] Xu Y, Luo BL, Jia R, Xiao J, Wang XQ, Yang YD, Xue S, Zeng ZH, Brown RW, Zang HD. 2024. Quantifying synergies and trade-offs in the food-energy-soil-environment nexus under organic fertilization. J Environ Manage 349:119526. doi:10.1016/j.jenvman.2023.119526 37956518

[7] Wang ZH, Yang TJ, Mei XL, Wang NQ, Li XG, Yang QS, Dong CX, Jiang GF, Lin J, Xu YC, Shen QR, Jousset A, Banerjee S. 2022. Bio-organic fertilizer promotes pear yield by shaping the rhizosphere microbiome composition and functions. Microbiol Spectr 10:14. doi:10.1128/spectrum.03572-22 PMC9769518 36453930

[8] Jiang YP, Li KR, Chen SF, Fu XL, Feng SY, Zhuang ZS. 2022. A sustainable agricultural supply chain considering substituting organic manure for chemical fertilizer. Sustain Prod Consum 29:432–446. doi:10.1016/j.spc.2021.10.025

[9] Kilic N. 2023. Synergistic effect of organic and biofertilizers on strawberry cultivation. Sustainability 15:8206. doi:10.3390/su15108206

[10] Shi YW, Niu XX, Chen BZ, Pu SH, Ma HH, Li P, Feng GP, Ma XW. 2023. Chemical fertilizer reduction combined with organic fertilizer affects the soil microbial community and diversity and yield of cotton. Front Microbiol 14:1295722. doi:10.3389/fmicb.2023.1295722 38053554 PMC10694218

[11] Ribeiro JC, Portz A, Dos Santos FS, Pereira MG, Zonta E. 2023. Oil well drill cuttings and sunflower cake: effects on sunflower crop and soil chemical attributes. Environ Technol 44:3342–3353. doi:10.1080/09593330.2022.2057238 35317710

[12] Mahalik JK, Mohapatra K, Sahu A. 2020. Efficacy of bacterial antagonists as seed treatment and neem oil cake against Meloidogyne incognita in cucumber (Cucumis sativus L.). Ann Plant Prot Sci 28:85. doi:10.5958/0974-0163.2020.00020.8

[13] Mazzoncini M, Antichi D, Tavarini S, Silvestri N, Lazzeri L, D’Avino L. 2015. Effect of defatted oilseed meals applied as organic fertilizers on vegetable crop production and environmental impact. Ind Crops Prod 75:54–64. doi:10.1016/j.indcrop.2015.04.061

[14] Bai HM, Liu MY, Jing YP, Li YJ, Chen SH, Xue GP, Wang JG, Suo QY, Jiang W. 2024. Effects of organic fertilizer with different degrees of maturity on bacteria in saline-alkali soil. Agronomy 14:1148. doi:10.3390/agronomy14061148

[15] Xie H, Wei Y, Yi C, Wang Y, Zhao Z, Liu X. 2023. Effects of organic fertilizers with different maturities on soil improvement and soybean yield. Agronomy 13:3004. doi:10.3390/agronomy13123004

[16] Senesi N. 1989. Composted materials as organic fertilizers. Sci Total Environ 81–82:521–542. doi:10.1016/0048-9697(89)90161-7

[17] Zhang YP, Ju SN, Wang WR, Wu FZ, Pan K. 2024. Effects of decomposed and undecomposed straw of three crops on clubroot disease of Chinese cabbage and soil nutrients. Sci Rep 14:22990. doi:10.1038/s41598-024-72899-7 39362893 PMC11449906

[18] Daur I, Saad MM, Eida AA, Ahmad S, Shah ZH, Ihsan MZ, Muhammad Y, Sohrab SS, Hirt H. 2018. Boosting alfalfa (Medicago sativa L.) production with rhizobacteria from various plants in Saudi Arabia. Front Microbiol 9:477. doi:10.3389/fmicb.2018.00477 29670582 PMC5893776

[19] Wang XB, Han QS. 2022. A closer examination of the 'abundant-center' for ectomycorrhizal fungal community associated with Picea crassifolia in China. Front Plant Sci 13:759801. doi:10.3389/fpls.2022.759801 35283884 PMC8908202

[20] Chen HY, Yu ST, Yu Z, Ma M, Liu MY, Pei HY. 2024. Phycoremediation potential of salt-tolerant microalgal species: motion, metabolic characteristics, and their application for saline-alkali soil improvement in eco-farms. Microorganisms 12:676. doi:10.3390/microorganisms12040676 38674620 PMC11052205

[21] Guo S, Xiong W, Hang XN, Gao ZL, Jiao ZX, Liu HJ, Mo YN, Zhang N, Kowalchuk GA, Li R, Shen QR, Geisen SF. 2021. Protists as main indicators and determinants of plant performance. Microbiome 9:64. doi:10.1186/s40168-021-01025-w 33743825 PMC7981826

[22] Wang JX, Gao J, Zhang HQ, Tang M. 2022. Changes in rhizosphere soil fungal communities of Pinus tabuliformis plantations at different development stages on the Loess Plateau. IJMS 23:6753. doi:10.3390/ijms23126753 35743198 PMC9223801

[23] Yu JT, Zhang XN, Xu CY, Hao MH, Choe C, He HJ. 2022. Thinning can increase shrub diversity and decrease herb diversity by regulating light and soil environments. Front Plant Sci 13:948648. doi:10.3389/fpls.2022.948648 35991461 PMC9389291

[24] Xiang XJ, Zhang JY, Li GL, Leng K, Sun LY, Qin WJ, Peng CR, Xu CX, Liu J, Jiang YJ. 2022. Positive feedback between peanut and arbuscular mycorrhizal fungi with the application of hairy vetch in Ultisol. Front Microbiol 13:1002459. doi:10.3389/fmicb.2022.1002459 36225353 PMC9549289

[25] Li JS, Zhao YQ, Shao XQ, Huang D, Shang JY, Li H, He YX, Liu KS. 2021. The mixed addition of biochar and nitrogen improves soil properties and microbial structure of moderate-severe degraded Alpine grassland in Qinghai-Tibet Plateau. Front Plant Sci 12:765041. doi:10.3389/fpls.2021.765041 34880889 PMC8647844

[26] Gu Y, Wang JH, Cai WJ, Li GL, Mei Y, Yang SH. 2021. Different amounts of nitrogen fertilizer applications alter the bacterial diversity and community structure in the rhizosphere soil of sugarcane. Front Microbiol 12:721441. doi:10.3389/fmicb.2021.721441 34616383 PMC8489880

[27] Huang P, Shen FY, Abbas A, Wang H, Du YZ, Du DL, Hussain S, Javed T, Alamri S. 2022. Effects of different nitrogen forms and competitive treatments on the growth and antioxidant system of Wedelia trilobata and Wedelia chinensis under high nitrogen concentrations. Front Plant Sci 13:851099. doi:10.3389/fpls.2022.851099 35401616 PMC8988914

[28] Chen SF, Zhou YQ, Chen YR, Gu J. 2018. Fastp: an ultra-fast all-in-one FASTQ preprocessor. Bioinformatics 34:i884–i890. doi:10.1093/bioinformatics/bty560 30423086 PMC6129281

[29] Magoč T, Salzberg SL. 2011. FLASH: fast length adjustment of short reads to improve genome assemblies. Bioinformatics 27:2957–2963. doi:10.1093/bioinformatics/btr507 21903629 PMC3198573

[30] Bokulich NA, Subramanian S, Faith JJ, Gevers D, Gordon JI, Knight R, Mills DA, Caporaso JG. 2013. Quality-filtering vastly improves diversity estimates from Illumina amplicon sequencing. Nat Methods 10:57–59. doi:10.1038/nmeth.2276 23202435 PMC3531572

[31] Edgar RC. 2010. Search and clustering orders of magnitude faster than BLAST. Bioinformatics 26:2460–2461. doi:10.1093/bioinformatics/btq461 20709691

[32] Edgar RC, Haas BJ, Clemente JC, Quince C, Knight R. 2011. UCHIME improves sensitivity and speed of chimera detection. Bioinformatics 27:2194–2200. doi:10.1093/bioinformatics/btr381 21700674 PMC3150044

[33] Pruesse E, Quast C, Knittel K, Fuchs BM, Ludwig W, Peplies J, Glöckner FO. 2007. SILVA: a comprehensive online resource for quality checked and aligned ribosomal RNA sequence data compatible with ARB. Nucleic Acids Res 35:7188–7196. doi:10.1093/nar/gkm864 17947321 PMC2175337

[34] Nilsson RH, Larsson K-H, Taylor AFS, Bengtsson-Palme J, Jeppesen TS, Schigel D, Kennedy P, Picard K, Glöckner FO, Tedersoo L, Saar I, Kõljalg U, Abarenkov K. 2019. The UNITE database for molecular identification of fungi: handling dark taxa and parallel taxonomic classifications. Nucleic Acids Res 47:D259–D264. doi:10.1093/nar/gky1022 30371820 PMC6324048

[35] Wang Q, Garrity GM, Tiedje JM, Cole JR. 2007. Naive Bayesian classifier for rapid assignment of rRNA sequences into the new bacterial taxonomy. Appl Environ Microbiol 73:5261–5267. doi:10.1128/AEM.00062-07 17586664 PMC1950982

[36] Segata N, Izard J, Waldron L, Gevers D, Miropolsky L, Garrett WS, Huttenhower C. 2011. Metagenomic biomarker discovery and explanation. Genome Biol 12. doi:10.1186/gb-2011-12-6-r60 PMC3218848 21702898

[37] Zhu X-Q, Chen Y, Jia M, Dai H-J, Zhou Y-B, Yang H-W, Zhou P, Du Y, Wang G, Bai Y-X, Wang N. 2024. Managing tobacco black shank disease using biochar: direct toxicity and indirect ecological mechanisms. Microbiol Spectr 12:e0014924. doi:10.1128/spectrum.00149-24 39212424 PMC11448098

[38] Ye L, Zhao X, Bao E, Li J, Zou Z, Cao K. 2020. Bio-organic fertilizer with reduced rates of chemical fertilization improves soil fertility and enhances tomato yield and quality. Sci Rep 10:177. doi:10.1038/s41598-019-56954-2 31932626 PMC6957517

[39] Huang A, Wang Z, Yang D, Yang S, Bai W, Wu N, Lu X, Liu Z. 2023. Effects of tea oil camellia (Camellia oleifera Abel.) shell-based organic fertilizers on the physicochemical property and microbial community structure of the rhizosphere soil. Front Microbiol 14. doi:10.3389/fmicb.2023.1231978 PMC10448393 37637109

[40] Duong TTT, Verma SL, Penfold C, Marschner P. 2013. Nutrient release from composts into the surrounding soil. Geoderma 195–196:42–47. doi:10.1016/j.geoderma.2012.11.010

[41] Zhu M, Xu D, Si G, Peng C, Yuan J, Zhao S. 2023. Effects of different organic fertilisers on the microbial functional diversity and bacterial communities in a tobacco soil. Arch Agron Soil Sci 69:1566–1578. doi:10.1080/03650340.2022.2103548

[42] Chen C, Zhang J, Lu M, Qin C, Chen Y, Yang L, Huang Q, Wang J, Shen Z, Shen Q. 2016. Microbial communities of an arable soil treated for 8 years with organic and inorganic fertilizers. Biol Fertil Soils 52:455–467. doi:10.1007/s00374-016-1089-5

[43] Chen D, Zhou Y, Wang M, Mujtaba Munir MA, Lian J, Yu S, Dai K, Yang X. 2022. Succession pattern in soil micro-ecology under tobacco (Nicotiana tabacum L.) continuous cropping circumstances in Yunnan Province of southwest China. Front Microbiol 12:785110. doi:10.3389/fmicb.2021.785110 35185815 PMC8851204

[44] Su Y, Zi HY, Wei XM, Hu BB, Deng XP, Chen Y, Jiang YL. 2022. Application of manure rather than plant-origin organic fertilizers alters the fungal community in continuous cropping tobacco soil. Front Microbiol 13:818956. doi:10.3389/fmicb.2022.818956 35516429 PMC9063659

[45] Yoneda Y, Yamamoto K, Makino A, Tanaka Y, Meng X-Y, Hashimoto J, Shin-Ya K, Satoh N, Fujie M, Toyama T, Mori K, Ike M, Morikawa M, Kamagata Y, Tamaki H. 2021. Novel plant-associated acidobacteria promotes growth of common floating aquatic plants, duckweeds. Microorganisms 9:15. doi:10.3390/microorganisms9061133 PMC8225144 34074043

[46] Lyons TW, Fike DA, Zerkle A. 2015. Emerging biogeochemical views of Earth's ancient microbial worlds. Elements 11:415–421. doi:10.2113/gselements.11.6.415

[47] Lidbury IDEA, Scanlan DJ, Murphy ARJ, Christie-Oleza JA, Aguilo-Ferretjans MM, Hitchcock A, Daniell TJ. 2022. A widely distributed phosphate-insensitive phosphatase presents a route for rapid organophosphorus remineralization in the biosphere. Proc Natl Acad Sci USA 119:e2118122119. doi:10.1073/pnas.2118122119 35082153 PMC8812569

[48] Fadiji AE, Ayangbenro AS, Babalola OO. 2020. Metagenomic profiling of the community structure, diversity, and nutrient pathways of bacterial endophytes in maize plant. Antonie Van Leeuwenhoek 113:1559–1571. doi:10.1007/s10482-020-01463-w 32803452

[49] Manici LM, Caputo F, De Sabata D, Fornasier F. 2024. The enzyme patterns of Ascomycota and Basidiomycota fungi reveal their different functions in soil. Appl Soil Ecol 196:105323. doi:10.1016/j.apsoil.2024.105323

[50] Zhou DY, Hou MY, Leng CY, Li RJ, Xing YM, Chen J. 2025. Deciphering the root microbiome and its relationship with active compound accumulation in medicinal Dendrobium officinale (Orchidaceae) from different regions. Ind Crops Prod 226:120692. doi:10.1016/j.indcrop.2025.120692

[51] Ren H, Deng Y, Zhao D, Jin W, Xie G, Peng B, Dai H, Wang B. 2024. Structures and diversities of bacterial communities in oil-contaminated soil at shale gas well site assessed by high-throughput sequencing. Environ Sci Pollut Res 31:10766–10784. doi:10.1007/s11356-023-31344-4 38200199

[52] Zheng GD, Cheng Y, Zhu YL, Yang JX, Wang L, Chen TB. 2022. Correlation of microbial dynamics to odor production and emission in full-scale sewage sludge composting. Bioresour Technol 360:127597. doi:10.1016/j.biortech.2022.127597 35835422

[53] Mishra S, Goyal D, Phurailatpam L. 2021. Targeted 16S rRNA gene and ITS2 amplicon sequencing of leaf and spike tissues of Piper longum identifies new candidates for bioprospecting of bioactive compounds. Arch Microbiol 203:3851–3867. doi:10.1007/s00203-021-02356-w 34013420

[54] O’Meara TR. 2024. Understanding pathogen emergence through the lens of Candida auris. Nat Microbiol 9:1399–1400. doi:10.1038/s41564-024-01700-2 38773313

[55] Tan X-M, Chen X-M, Wang C-L, Jin X-H, Cui J-L, Chen J, Guo S-X, Zhao L-F. 2012. Isolation and identification of endophytic fungi in roots of nine Holcoglossum plants (Orchidaceae) collected from Yunnan, Guangxi, and Hainan provinces of China. Curr Microbiol 64:140–147. doi:10.1007/s00284-011-0045-8 22057921

[56] Huang B, Jia H, Han X, Gou J, Huang C, Wang J, Wei J, Wang J, Zhang C. 2021. Effects of biocontrol Bacillus and fermentation bacteria additions on the microbial community, functions and antibiotic resistance genes of prickly ash seed oil meal-biochar compost. Bioresour Technol 340:125668. doi:10.1016/j.biortech.2021.125668 34339999

[57] Zhang L, Liu Z, Wang Y, Zhang J, Wan S, Huang Y, Yun T, Xie J, Wang W. 2022. Biocontrol potential of endophytic Streptomyces malaysiensis 8ZJF-21 from medicinal plant against banana fusarium wilt caused by Fusarium oxysporum f. sp. cubense tropical race 4. Front Plant Sci 13. doi:10.3389/fpls.2022.874819 PMC9131080 35646017

[58] Schwarze FWMR, Gholam Z, Nyström G, Heeb M. 2022. Microfibrillated cellulose as growth media for cultivation and maintenance of wood decay fungi. J Microbiol Methods 203:106615. doi:10.1016/j.mimet.2022.106615 36356691

[59] Gonçalves OS, Fernandes AS, Tupy SM, Ferreira TG, Almeida LN, Creevey CJ, Santana MF. 2024. Insights into plant interactions and the biogeochemical role of the globally widespread Acidobacteriota phylum. Soil Biol Biochem 192:109369. doi:10.1016/j.soilbio.2024.109369

[60] Tan YL, Wang J, He YG, Yu XM, Chen SJ, Penttinen P, Liu SL, Yang Y, Zhao K, Zou LK. 2023. Organic fertilizers shape soil microbial communities and increase soil amino acid metabolites content in a blueberry orchard. Microb Ecol 85:232–246. doi:10.1007/s00248-022-01960-7 35064809

