# Supplementary material for: Composted organic manures modulated soil–microbe interactions that enhanced the growth of tobacco by improving rhizospheric microbial structure and soil nutrients
Source: Microbiol Spectr. 2026 Apr 16;14(6):e02586-25. doi: 10.1128/spectrum.02586-25 (PMC13228057; doi:10.1128/spectrum.02586-25)
Supplement: Supplemental tables — Tables S1 and S2. [file spectrum.02586-25-s0001.docx]

Table S1 LEfSe Analysis bacterial differential microbes

| Family | LDA score | Treatment |
| --- | --- | --- |
| Crocinitomicaceae | 4.10736073 | CK |
| Cellvibrionaceae | 4.05631957 | CK |
| Gemmatimonadaceae | 3.08498605 | CK |
| Sphingomonadaceae | 3.12877539 | CK |
| Oxalobacteraceae | 3.07498753 | CK |
| Chitinophagaceae | 2.98321976 | CK |
| Nitrosomonadaceae | 2.43383858 | CK |
| Sphingomonadaceae | 2.64603293 | CK |
| Caulobacteraceae | 2.56103943 | CK |
| Anaerolineaceae | 2.41693517 | CK |
| Xanthobacteraceae | 2.38322816 | CK |
| Verrucomicrobiaceae | 2.33516119 | CK |
| Sphingomonadaceae | 2.2457274 | CK |
| Sphingomonadaceae | 2.22180979 | CK |
| Silvanigrellaceae | 2.07369115 | CK |
| Peptostreptococcaceae | 2.0600647 | CK |
| Lactobacillaceae | 2.14164221 | CK |
| Hymenobacteraceae | 2.04478258 | CK |
| Syntrophobacteraceae | 2.00468747 | CK |
| Hymenobacteraceae | 2.18287706 | CK |
| Sporomusaceae | 2.25706152 | CK |
| Methylophilaceae | 2.22259741 | CK |
| Nocardioidaceae | 4.6938727 | MZ0 |
| Enterobacteriaceae | 4.58204713 | MZ0 |
| Microbacteriaceae | 4.338341 | MZ0 |
| Chitinophagaceae | 4.13889339 | MZ0 |
| Polyangiaceae | 3.90113305 | MZ0 |
| Bacillaceae | 3.8357983 | MZ0 |
| Cellulomonadaceae | 3.59381266 | MZ0 |
| Geodermatophilaceae | 3.57797546 | MZ0 |
| Ferrovibrionaceae | 3.57222744 | MZ0 |
| Parachlamydiaceae | 3.52042962 | MZ0 |
| Chitinophagaceae | 3.44772807 | MZ0 |
| Pseudonocardiaceae | 3.38508949 | MZ0 |
| Azospirillaceae | 3.38501847 | MZ0 |
| Terrimicrobiaceae | 3.33329814 | MZ0 |
| Enterobacteriaceae | 3.3248914 | MZ0 |
| Chitinophagaceae | 3.25969467 | MZ0 |
| Sphingomonadaceae | 3.21361592 | MZ0 |
| Legionellaceae | 3.20637529 | MZ0 |
| Rhodanobacteraceae | 3.14806009 | MZ0 |
| Rhodocyclaceae | 3.11620435 | MZ0 |
| Hahellaceae | 3.10677946 | MZ0 |
| Alcaligenaceae | 3.07143226 | MZ0 |
| Alcaligenaceae | 2.99450672 | MZ0 |
| Clostridiaceae | 2.95927113 | MZ0 |
| Polyangiaceae | 2.95381162 | MZ0 |
| Enterobacteriaceae | 2.94592598 | MZ0 |
| Opitutaceae | 2.93384892 | MZ0 |
| Unknown_Family | 2.89266303 | MZ0 |
| Propionibacteriaceae | 2.84578556 | MZ0 |
| Clostridiaceae | 2.83172748 | MZ0 |
| Moraxellaceae | 2.77338913 | MZ0 |
| Planococcaceae | 2.76360135 | MZ0 |
| Sphingobacteriaceae | 2.72185717 | MZ0 |
| Tepidisphaeraceae | 2.71833132 | MZ0 |
| Saccharimonadaceae | 2.69065721 | MZ0 |
| Sphingobacteriaceae | 2.68687841 | MZ0 |
| Brevibacillaceae | 2.67267573 | MZ0 |
| Coleofasciculaceae | 2.59224339 | MZ0 |
| Xanthobacteraceae | 2.55713157 | MZ0 |
| Alcaligenaceae | 2.55207627 | MZ0 |
| Dongiaceae | 2.55031714 | MZ0 |
| Beijerinckiaceae | 2.51346231 | MZ0 |
| Diplorickettsiaceae | 2.40028434 | MZ0 |
| Streptomycetaceae | 2.38642692 | MZ0 |
| Spirosomaceae | 2.37754054 | MZ0 |
| Brevibacteriaceae | 2.37380384 | MZ0 |
| Rhizobiaceae | 2.34272272 | MZ0 |
| Hyphomonadaceae | 2.31376866 | MZ0 |
| Sphingobacteriaceae | 2.29040431 | MZ0 |
| Promicromonosporaceae | 2.25441111 | MZ0 |
| Sphingobacteriaceae | 2.14704697 | MZ0 |
| Alcaligenaceae | 2.14543788 | MZ0 |
| Myxococcaceae | 2.12827533 | MZ0 |
| Comamonadaceae | 2.03623973 | MZ0 |
| Rhodanobacteraceae | 1.79581275 | MZ0 |
| Micromonosporaceae | 1.67208962 | MZ0 |
| Abditibacteriaceae | 1.66533029 | MZ0 |
| Burkholderiaceae | 1.64229142 | MZ0 |
| Microbacteriaceae | 1.55030082 | MZ0 |
| Polyangiaceae | 3.75579609 | MZ1 |
| Xanthomonadaceae | 3.56615287 | MZ1 |
| Beijerinckiaceae | 3.2578827 | MZ1 |
| Sporolactobacillaceae | 3.13633387 | MZ1 |
| Unknown_Family | 3.13578601 | MZ1 |
| Beijerinckiaceae | 3.0229244 | MZ1 |
| Rubritaleaceae | 2.99100153 | MZ1 |
| Cytophagaceae | 2.89379438 | MZ1 |
| Bacillaceae | 2.86294828 | MZ1 |
| Bdellovibrionaceae | 2.72781021 | MZ1 |
| Myxococcaceae | 2.64543067 | MZ1 |
| Alcaligenaceae | 2.59557034 | MZ1 |
| Anaerolineaceae | 2.47099133 | MZ1 |
| Thermomonosporaceae | 2.26496212 | MZ1 |
| Paenibacillaceae | 1.44490848 | MZ1 |
| Deinococcaceae | 1.44201026 | MZ1 |
| Rhodothermaceae | 1.4230247 | MZ1 |
| Rhodocyclaceae | 4.53901143 | MZ2 |
| Bacteriovoracaceae | 4.12298723 | MZ2 |
| Oxalobacteraceae | 3.99305305 | MZ2 |
| Nitrosomonadaceae | 3.73582192 | MZ2 |
| SC_I_84 | 3.63348942 | MZ2 |
| Chitinophagaceae | 3.56055421 | MZ2 |
| Comamonadaceae | 3.55046428 | MZ2 |
| Rhodocyclaceae | 3.49991038 | MZ2 |
| Planococcaceae | 3.40665118 | MZ2 |
| Oxalobacteraceae | 3.33481485 | MZ2 |
| Rubinisphaeraceae | 3.32032515 | MZ2 |
| Magnetospirillaceae | 3.31725039 | MZ2 |
| Sphingobacteriaceae | 3.24337106 | MZ2 |
| Weeksellaceae | 3.22959138 | MZ2 |
| Sphingomonadaceae | 3.21077401 | MZ2 |
| Alcaligenaceae | 3.19331882 | MZ2 |
| Phycisphaeraceae | 3.17256496 | MZ2 |
| Comamonadaceae | 3.15080752 | MZ2 |
| Blastocatellaceae | 3.14653026 | MZ2 |
| Pirellulaceae | 3.1255144 | MZ2 |
| Blastocatellaceae | 3.09318534 | MZ2 |
| Blastocatellaceae | 3.00317848 | MZ2 |
| Opitutaceae | 2.99334995 | MZ2 |
| Herpetosiphonaceae | 2.97798371 | MZ2 |
| Xanthomonadaceae | 2.93928057 | MZ2 |
| Syntrophobacteraceae | 2.86887503 | MZ2 |
| A4b | 2.82763887 | MZ2 |
| Comamonadaceae | 2.81619913 | MZ2 |
| Spirosomaceae | 2.65944031 | MZ2 |
| Chthoniobacteraceae | 2.60938332 | MZ2 |
| Chitinophagaceae | 2.58424685 | MZ2 |
| Desulfobulbaceae | 2.57229682 | MZ2 |
| Unknown_Family | 2.50897944 | MZ2 |
| Thermoanaerobaculaceae | 2.49506352 | MZ2 |
| Planococcaceae | 2.22106448 | MZ2 |
| Rhizobiales_Incertae_Sedis | 1.82024039 | MZ2 |
| Comamonadaceae | 1.67367879 | MZ2 |
| Oxalobacteraceae | 1.61126747 | MZ2 |
| Rhodanobacteraceae | 1.34626711 | MZ2 |
| Xanthomonadaceae | 1.29441821 | MZ2 |
| Xanthomonadaceae | 1.21584311 | MZ2 |
| Rhodanobacteraceae | 1.0114298 | MZ2 |
| Bacillaceae | 4.07330579 | MZ3 |
| Streptosporangiaceae | 4.01286607 | MZ3 |
| Peptostreptococcaceae | 3.87649217 | MZ3 |
| Mycobacteriaceae | 3.80829874 | MZ3 |
| Rhodanobacteraceae | 3.7882697 | MZ3 |
| Thermomonosporaceae | 3.57877568 | MZ3 |
| Unknown_Family | 3.40361141 | MZ3 |
| Comamonadaceae | 3.28335478 | MZ3 |
| Nitrosomonadaceae | 3.26771898 | MZ3 |
| Chitinophagaceae | 3.24775997 | MZ3 |
| Sporichthyaceae | 3.18230641 | MZ3 |
| Flavobacteriaceae | 3.13103946 | MZ3 |
| Blastocatellaceae | 3.07808483 | MZ3 |
| Chitinophagaceae | 3.00808972 | MZ3 |
| Steroidobacteraceae | 2.90371955 | MZ3 |
| Halomonadaceae | 2.89930408 | MZ3 |
| Nocardioidaceae | 2.87375481 | MZ3 |
| Thermoactinomycetaceae | 2.79108741 | MZ3 |
| Sphingobacteriaceae | 2.75026807 | MZ3 |
| Opitutaceae | 2.50273392 | MZ3 |
| Schlesneriaceae | 2.48449817 | MZ3 |
| Xanthobacteraceae | 2.37070636 | MZ3 |
| Enterobacteriaceae | 2.29997842 | MZ3 |
| Sphingobacteriaceae | 2.21224758 | MZ3 |
| Caulobacteraceae | 1.94707225 | MZ3 |
| Pirellulaceae | 1.72694972 | MZ3 |
| Sphingomonadaceae | 1.68148557 | MZ3 |
| Chthoniobacteraceae | 1.28638017 | MZ3 |
| Cytophagaceae | 1.13446434 | MZ3 |

Table S2 LEfSe Analysis fungal differential microbes

| Family | LDA score | Treatment |
| --- | --- | --- |
| Cucurbitariaceae | 3.69 | CK |
| Chaetopeltidaceae | 2.09 | CK |
| Didymellaceae | 2.26 | CK |
| Glomeraceae | 2.08 | CK |
| Cordycipitaceae | 2.42 | CK |
| Nectriaceae | 2.11 | CK |
| Psathyrellaceae | 2.47 | CK |
| Meruliaceae | 2.31 | CK |
| Microdochiaceae | 2.99 | CK |
| Microascaceae | 2.32 | CK |
| Spizellomycetaceae | 3.93 | CK |
| Diversisporaceae | 2.54 | CK |
| Pleosporaceae | 2.46 | CK |
| Strophariaceae | 2.12 | CK |
| Phaeosphaeriaceae | 2.60 | CK |
| Halteriidae | 3.37 | CK |
| Lasiosphaeriaceae | 3.79 | CK |
| Sordariales_fam_Incertae_sedis | 2.43 | CK |
| Nectriaceae | 3.70 | CK |
| Herpotrichiellaceae | 2.42 | CK |
| Hypocreales_fam_Incertae_sedis | 2.42 | CK |
| Phaeosphaeriaceae | 3.13 | CK |
| Onygenales_fam_Incertae_sedis | 2.56 | CK |
| Stachybotryaceae | 2.72 | CK |
| Nectriaceae | 2.99 | CK |
| Clavicipitaceae | 2.26 | CK |
| Bolbitiaceae | 4.50 | CK |
| Brassicaceae | 2.19 | CK |
| Hysterangiaceae | 3.15 | CK |
| Morosphaeriaceae | 2.59 | CK |
| Pleosporaceae | 2.58 | CK |
| Choanephoraceae | 2.21 | CK |
| Hypocreales_fam_Incertae_sedis | 2.35 | CK |
| Periconiaceae | 2.34 | CK |
| Bracteacoccaceae | 2.46 | CK |
| Solanaceae | 5.01 | CK |
| Ustilaginaceae | 2.32 | CK |
| Asteraceae | 2.98 | CK |
| Aspergillaceae | 3.12 | CK |
| Scenedesmaceae | 3.96 | CK |
| Sympoventuriaceae | 2.18 | CK |
| Nectriaceae | 2.21 | CK |
| Scenedesmaceae | 2.04 | CK |
| Arthrodermataceae | 2.60 | CK |
| Nectriaceae | 2.45 | CK |
| Chaetophoraceae | 2.03 | CK |
| Microbotryaceae | 2.26 | CK |
| Nectriaceae | 3.37 | CK |
| Hypocreaceae | 2.12 | CK |
| Meloidogynidae | 2.13 | CK |
| Powellomycetaceae | 2.59 | CK |
| Cladophoraceae | 2.74 | CK |
| Lamiaceae | 3.18 | CK |
| Microascaceae | 3.07 | CK |
| Nectriaceae | 4.79 | CK |
| Mucoraceae | 3.74 | CK |
| Gloniaceae | 2.30 | CK |
| Bionectriaceae | 2.52 | CK |
| Scenedesmaceae | 2.53 | CK |
| Lasiosphaeriaceae | 3.34 | CK |
| Chlorococcaceae | 2.51 | CK |
| Sanchytriaceae | 2.88 | CK |
| Prasiolaceae | 2.20 | CK |
| Glomeraceae | 2.22 | CK |
| Pottiaceae | 2.27 | CK |
| Pleosporaceae | 2.80 | CK |
| Chaetomiaceae | 3.32 | CK |
| Cannabaceae | 3.44 | CK |
| Hypocreales_fam_Incertae_sedis | 2.10 | CK |
| Trebouxiaceae | 2.40 | CK |
| Stachybotryaceae | 2.69 | CK |
| Bolbitiaceae | 2.86 | MZ0 |
| Chaetomiaceae | 4.97 | MZ0 |
| Malvaceae | 3.61 | MZ0 |
| Dipodascaceae | 3.64 | MZ0 |
| Rhizopodaceae | 3.82 | MZ0 |
| Pyronemataceae | 2.62 | MZ0 |
| Bionectriaceae | 2.60 | MZ0 |
| Aspergillaceae | 4.51 | MZ0 |
| Pleurotheciaceae | 2.48 | MZ0 |
| Basidiobolaceae | 2.23 | MZ1 |
| Vorticellidae | 2.66 | MZ1 |
| Asteraceae | 3.25 | MZ1 |
| Polygonaceae | 2.97 | MZ1 |
| Magnaporthaceae | 2.27 | MZ1 |
| Chaetomiaceae | 2.60 | MZ1 |
| Trichosporonaceae | 3.14 | MZ1 |
| Erythrobasidiaceae | 3.42 | MZ1 |
| Pezizaceae | 2.59 | MZ1 |
| Vahlkampfiidae | 2.43 | MZ1 |
| Tricholomataceae | 2.88 | MZ1 |
| Chaetomiaceae | 3.83 | MZ1 |
| Ustilaginaceae | 2.25 | MZ1 |
| Trichosporonaceae | 2.49 | MZ1 |
| Trachelostylidae | 2.03 | MZ1 |
| Cephalobidae | 3.95 | MZ1 |
| Hydnangiaceae | 2.78 | MZ1 |
| Ceratobasidiaceae | 4.29 | MZ1 |
| Bolbitiaceae | 3.41 | MZ2 |
| Saccharomycetales_fam_Incertae_sedis | 2.64 | MZ2 |
| Rhizophlyctidaceae | 3.13 | MZ2 |
| Lentitheciaceae | 2.73 | MZ2 |
| Agaricaceae | 2.97 | MZ2 |
| Cantharellales_fam_Incertae_sedis | 2.74 | MZ2 |
| Saccharomycetaceae | 2.20 | MZ2 |
| Serendipitaceae | 3.23 | MZ2 |
| Psathyrellaceae | 2.85 | MZ2 |
| Piskurozymaceae | 2.29 | MZ2 |
| Helotiales_fam_Incertae_sedis | 2.69 | MZ2 |
| Mortierellaceae | 4.44 | MZ2 |
| Ascobolaceae | 4.16 | MZ2 |
| Pyronemataceae | 3.28 | MZ3 |
| Russulaceae | 3.21 | MZ3 |
| Thermoascaceae | 2.48 | MZ3 |
| Microascaceae | 2.50 | MZ3 |
| Myrmecridiaceae | 2.16 | MZ3 |
| Sporormiaceae | 4.59 | MZ3 |
| Chaetomiaceae | 3.85 | MZ3 |
| Lasiosphaeriaceae | 4.36 | MZ3 |
| Orbiliales_fam_Incertae_sedis | 2.35 | MZ3 |
| Pezizales_fam_Incertae_sedis | 3.48 | MZ3 |
| Pleurotheciaceae | 2.24 | MZ3 |
| Biatriosporaceae | 2.28 | MZ3 |
| Clavariaceae | 3.43 | MZ3 |
| Lasiosphaeriaceae | 2.24 | MZ3 |
